# Molecular characterization of virulence genes of *Streptococcus equi* subsp. *equi* and *Streptococcus equi* subsp. *zooepidemicus* in equines

**DOI:** 10.14202/vetworld.2016.875-881

**Published:** 2016-08-19

**Authors:** R. Javed, A. K. Taku, Rakhi Gangil, R. K. Sharma

**Affiliations:** 1 Department of Microbiology, Faculty of Veterinary Sciences & Animal Husbandry, R.S. Pura, Jammu, Jammu and Kashmir, India; 2 Department of Microbiology, College of Veterinary Science and Animal Husbandry, Mhow, Madhya Pradesh, India

**Keywords:** polymerase chain reaction, *Streptococcus equi* sub sp. *equi*, and *Streptococcus equi* sub sp. *zooepidemicus*

## Abstract

**Aim:**

The aim was to determine the occurrence of streptococci in equines in Jammu (R. S. Pura, Katra), characterization of *Streptococci equi* subsp. *equi* and *Streptococcus equi* subsp. *zooepidemicus* with respect to their virulence traits and to determine antibiotic sensitivity pattern of virulent *Streptococcus* isolates.

**Materials and Methods:**

A total of 96 samples were collected from both clinically affected animals (exhibiting signs of respiratory tract disease) and apparently healthy animals and were sent to laboratory. The organisms were isolated on Columbia nalidixic acid agar containing 5% sheep blood as well as on sheep blood agar and confirmed by cultural characteristics and biochemical tests. Molecular detection of *Streptococcus* was done directly from cultures using *sodA* and *seM* gene-based polymerase chain reaction (PCR). Antibiogram was performed against five antibiotics such as amoxicillin, penicillin G, streptomycin, rifampicin, and methicillin.

**Results:**

During this study, a total 40 streptococcal isolates were obtained out of which 2 isolates were of *S. equi* subsp. *equi*, 12 isolates were from *S. equi* subsp. *zooepidemicus*. In the PCR-based detection, we revealed amplicons of 235 bp and 679 bp for confirmation of *sodA* and *seM* gene, respectively. In antibiogram, two isolates of *S. equi* subsp. *equi* were found resistant to penicillin G, and all other isolates were found sensitive to amoxicillin and streptomycin.

**Conclusion:**

The majority of streptococcal infections was due to *S. equi* subsp. *Zooepidemicus*, and thus was recognized as a potential pathogen of diseases of equines besides *S. equi* subsp. *equi*.

## Introduction

Equines play an important role in the socio-economic life of human population. They have been playing an important role in tourism promotion. Equines are used for working purposes, sports, and leisure activities, and nowadays, as means of transportation in the tourism industry. In Jammu and Kashmir, equine population constitutes the most preferred and economic means of transport used by tourists to visit various high altitude places including various places of religious importance. The total equine population in India is approximately 5.3 million, out of which 2.39 million (13.9%) is present in Jammu and Kashmir which stands at second position [1]. Upper respiratory tract infections are common in horses and can be caused by viral, fungal, and bacterial pathogens. Although a variety of bacterial agents have been associated with the respiratory problems in equines, the most important ones which are associated with upper respiratory tract diseases include streptococci (*Streptococcus equi* subsp. *equi* and *S. equi* subsp. *zooepidemicus*) and *Rhodococcus equi* [2]. Bacteria belonging to the genus *Streptococcus* are Gram-positive cocci that form chains or occur in pairs. Streptococci can be host specific or be transmitted between, and cause disease in, several species including zoonotic transmission to humans [3,4]. Molecular typing methods showed that human and equine isolates of *Streptococcus zooepidemicus* were identical or closely related; it emphasize that transmitted from horses can lead to severe infections in humans [5].

The most commonly isolated beta-hemolytic streptococci from horses with respiratory and genital diseases are *S. equi* subsp. *equi* and *S. equi* subsp. *zooepidemicus* [4]. *S. equi* subsp. *equi* is the most notorious agent associated with great economic losses to equine husbandry by affecting the pulmonary infections and reducing their performance. *S. equi* subsp. *zooepidemicus* is the ancestor of *S. equi* and is generally considered an opportunistic commensal of the equine upper respiratory tract [6,7]. *S. equi* spp. *zooepidemicus* is associated with a wide variety of infections in many animal species including horses, cows, swine, sheep, and dogs [8,9]. *S. equi* spp. *zooepidemicus* is most frequently isolated from the cases of equine pneumonia and pleuropneumonia [10], but it is also associated with infectious endometritis in the mare [11]. Upper respiratory disease caused by *S. zooepidemicus* can mimic mild cases of strangles [12], and the subspecies can also be isolated from horses with confirmed *S. equi* infection [13].

Most of the respiratory diseases are contagious; therefore, there is a great demand by clinicians and horse owners for earlier laboratory confirmation. Unfortunately, very less research has been done in India despite the high population in equines and their importance. Keeping these facts in view, this study was envisaged with the objective of determination of the prevalence of different species streptococci (*S. equi* subsp. *zooepidemicus* and *S. equi* subsp. *equi*) and characterization of their virulence factors and antibiotic sensitivity patterns.

## Materials and Methods

### Ethical approval

The approval from the Institutional Animal Ethics Committee (IAHC) to carry out this study was not required as no invasive technique was used. Nasal swab samples were being collected from clinically affected animals and healthy animals for this study as per standard collection procedure.

### Sample collection

The present investigation was conducted during August 2013 to July 2014. Samples were collected from R. S. Pura and Katra regions of Jammu (Jammu and Kashmir). A total of 96 nasal swab samples were collected aseptically from 50 clinically affected animals (exhibiting signs of respiratory tract disease) and 46 apparently healthy animals up to 6 months age. The samples were immediately transported to the laboratory of Department of Veterinary Microbiology and Immunology, Faculty of Veterinary Sciences and Animal Husbandry, Sher-e-Kashmir University of Agricultural Sciences and Technology of Jammu, R. S. Pura for further processing.

### Isolation and biochemical characterization of bacteria

Nasal swab samples were inoculated in Todd Hewitt broth and brain heart infusion (BHI) broth for enrichment at 37°C for 4 h. From these enrichment broths, the samples were inoculated on blood agar plates (containing 5% sheep blood) and Columbia nalidixic agar plates and incubated at 37°C for 48 h for the isolation of streptococci. The bacterial isolates which showed smooth translucent, shiny colonies with zones of β-hemolysis were selected for further processing. Pure colonies of bacteria were obtained by sub-culturing (2-3 times) on the 5% sheep blood agar. The bacterial isolates were presumptively identified based on color and status of hemolysis and Gram’s staining. All the Streptococcal isolates obtained were subjected to biochemical characterization using Histrep Identification Kit (KB 005A, Himedia, Mumbai, India). The kit contains 12 biochemical tests which include Voges-Proskauer, esculin hydrolysis, pyrrolidonyl arylamidase test, O-nitrophenyl-β-d-galactopyranoside, arginine utilization, and fermentation of seven sugars, *viz*., glucose, lactose, arabinose, sucrose, sorbitol, mannitol, and raffinose.

### Molecular detection of Streptococcus by polymerase chain reaction (PCR)

Species-specific PCR was used for detection of important bacterial pathogens from bacterial isolates targeting *16SrRNA* genus-specific PCR for *Streptococcus*, *SeM* gene for *S. equi* subsp. *equi* (*S. equi* subsp. *equi*), *sodA* gene for *S. equi* subsp. *zooepidemicus* (*S. equi* subsp. *zooepidemicus*), and *16S rRNA*. *SeM* gene encodes for the M like protein exclusively found in *S. equi* subsp. *equi*, which protects it from phagocytosis and renders it highly pathogenic while *sodA* gene encodes for superoxide dismutase enzyme which neutralizes the effect of superoxides by the host thereby protecting it from getting killed by neutrophils. *SeM* gene was amplified by PCR with light modification in given protocol [14]. PCR was carried out in a final reaction volume of 25 µl using 0.2 ml thin wall sterile and nuclease free PCR tubes. The PCR mixture contained a final concentration of 1.5 mM MgCl_2_, 0.20 mM concentrations of each 2’-deoxynucleoside 5’-triphosphate (dNTPs), 2.5 µl of ×10 PCR buffer, 1.0 µΜ of forward and reverse primers, 1.0 U of *Taq* DNA polymerase (Promega limited U.S.A), and 3.0 µl template DNA. The primers used in this study were procured from Chromous Biotech Pvt. Ltd., Bengaluru, India. The oligonucleotide primers used in the present study and predicted size of the PCR amplicon according to the previous study (Table-1) [15].

**Table-1:** List of oligonucleotide primers for detection of *SeM* gene (Timoney and Artiuschin, 1997).

Primer name	Nucleotide sequence	Product size (bp)
Forward primer (*SeM* 7)	5’ TGCATAAAGAAGTTCCTGTC 3’	679
Reverse primer (*SeM* 6)	5’ GATTCGGTAAGAGCTTGACG 3’	

Amplification was carried out in a thermal cycler (Eppendorf Mastercycler Gradient, Germany). The amplification cycle consisted of initial denaturation at 94°C for 2 min, followed by 35 cycles, each consisting of initial denaturation at 94°C for 10 s, annealing at 56°C for 10 s and extension at 72°C for 5 s which was followed by final extension at 72°C for 5 min. *sodA* gene was amplified by PCR with slight modifications [16]. PCR was carried out in a final reaction volume of 25 µl in 0.2 ml thin wall sterile and nuclease free PCR tubes (Eppendorf, Germany). The PCR mixture contained a final concentration of 1.5 mM MgCl_2_, 0.20 mM concentrations of each dNTPs, 2.5 µl of ×10 PCR buffer, 1.0 µΜ of forward and reverse primers, 3.00 µl template DNA and 1.0 U of *Taq* DNA polymerase (Promega Limited, USA). Primer sequences used in the study and predicted size of the PCR amplicon according to the previous study (Table-2) [16]. Amplification was carried out in a thermal cycler (Eppendorf Mastercycler Gradient, Germany) in the similar manner as that for *S. equi* subsp. *equi*. The confirmation of PCR product was done by electrophoresis of amplified products in 1.0% agarose gel in horizontal electrophoresis unit (Biometra, Germany). This gel was visualized under BioDocAnalyze (Biometra) and photographed. Molecular sizes at of standard molecular size marker.

**Table-2:** List of oligonucleotide primers for detection of *sodA* gene (Alber *et al.*, 2004).

Primer name	Nucleotide sequence	Product size (bp)
Forward primer	5’-CAGCATTCCTGCTGACATTCGTCAGG 3’	235
Reverse primer	5’-CTGACCAGCATTATTCACAACCAGCC 3’	

### Antimicrobial sensitivity assay

All the isolates of *S. equi* subsp. *equi*, *S. zooepidemicus*, and *R. equi* were subjected to antibiotic sensitivity test by disc diffusion method [17] using five antimicrobials, amoxiciin – 30 µg, penicillin G - 25 U, amikacin - 30 µg, streptomycin - 10 µg, and methicillin - 5 µg (Himedia, Mumbai, India). The antibiotic sensitivity assay was performed on 5% sheep blood agar. Two to three bacterial colonies were picked from culture plate and inoculated in the BHI and incubated at 37°C for 6 h. A sterile cotton swab was dipped in the BHI, and swab was inoculated by lawn method on 5% sheep blood agar. The test antibiotic discs were dispensed by antibiotic disc dispenser (Himedia, Mumbai, India).

## Results

**Figure-1:**
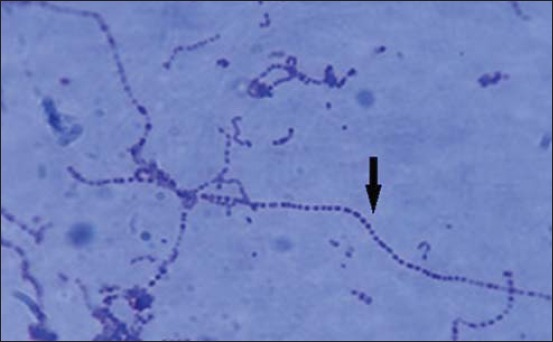
Long chains of *Streptococcus equi* subsp. *equi* Gram-staining.

**Figure-2:**
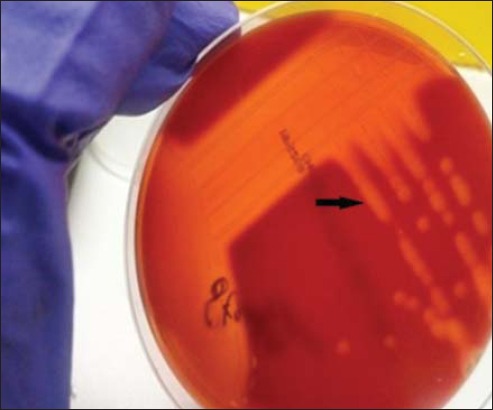
*Streptococcus equi* showing beta hemolysis on blood agar.

In this study, out of 96 samples (50 from diseased and 46 apparently healthy), total 218 bacterial isolates were obtained. From these bacterial isolates, 121 recovered from diseased animal and 97 from healthy animals. The majority of bacteria isolated (97 out of 121) from diseased animals were Gram-positive while others were Gram-negative. Similarly, 63 isolates from apparently healthy animals were Gram-positive while 34 were Gram-negative. Streptococci were presumptively identified as small, smooth, shiny, and hemolytic colonies (α/β) on 5% sheep blood agar and were purified by repeated sub-culturing on 5% sheep blood agar (Figure-1). Gram’s staining of these isolates revealed Gram-positive cocci in chain (Figure-2). 40 isolates of streptococci were identified based on cultural characteristics and Gram-staining was subjected to a series of biochemical tests for further characterization of the isolates. All the 40 streptococcal isolates were subjected to genus-specific 16S rRNA PCR for confirmation as streptococci. Out of 40 isolates, 20 were confirmed as belonging to genus *Streptococcus*. Out of these 20 isolates, 16 belonged to diseased animals while four were from apparently healthy animals. Confirmed streptococcal isolates were further subjected to species-specific PCR-based on virulence genes.

**Figure-3:**
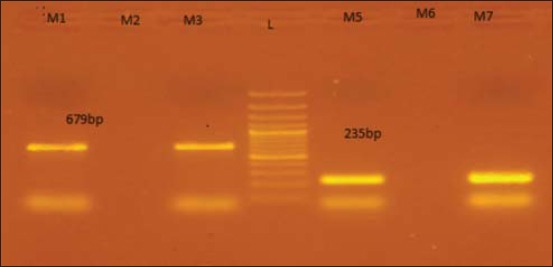
Amplified product of *Streptococcus equi* and *Streptococcus zooepidemicus*. Lane M1, M3: Amplified product of *S. equi* for *SeM* gene at 679 bp, Lane M2, M6: Negative sample, Lane M5, M7: Amplified product of *S. zooepidemicus* for *sodA* gene at 235 bp, Lane L: Molecular weight marker of 100 bp, (Himedia India).

All the 20 streptococcal isolates confirmed by 16S rRNA genus-specific primers were subjected to *SeM* PCR for detection of *S. equi* subsp. *equi*, and 2 (4.25%) an amplicon of 679 bp confirmatory for *S. equi* subsp. *equi* and other isolates were subjected to PCR for detection of *sodA* gene of *S. equi* subsp. *zooepidemicus* which revealed an amplicon of 235 bp confirmatory for *S. equi* subsp. *zooepidemicus* (Figure-3). PCR based results for *S. equi* subsp. *equi* and *S. equi* subsp. *zooepidemicus* from apparently healthy and diseased equines is given in Tables-3 and 4.

**Table-3:** Distribution of *S. equi* subsp. *equi* as detected by amplification of *SeM* gene from apparently healthy and diseased equines.

Region	Diseased animals		Apparently healthy animals		Total	
		
	Number of isolates as detected from 16SrRNA	Positive for *SeM* gene	Number of isolates as detected from 16SrRNA	Positive for *SeM* gene	Number of isolates as detected from 16SrRNA	Positive for *SeM* gene
R.S. Pura	4	0	1	0	5	0
Katra	12	2	3	0	15	2
Total	16	2	4	0	20	2

S. equi=Streptococcus equi

**Table-4:** PCR-based distribution of *sodA* gene from apparently healthy and diseased equines.

Region	Diseased		Apparently healthy		Total	
		
	Isolates subjected to PCR	Positive for *sodA* gene	Isolates subjected to PCR	Positive for *sodA* gene	Isolates subjected to PCR	Positive for *sodA* gene
R. S. Pura	4	2	1	1	5	3
Katra	10	8	3	1	13	9
Total	14	10	4	2	18	12

PCR=Polymerase chain reaction

The summary of the results of antibiotic sensitivity of two isolates of *S. equi* ssp. *equi* are shown in Table-5. The results revealed that amoxicillin and rifampicin were the most effective followed by streptomycin while maximum resistance was noted against penicillin G. Both the streptococcal isolates demonstrated intermediate zones with methicillin. The results of antibiotic sensitivity assay of 12 isolates of *S. equi* ssp. *zooepidemicus* are presented in Table-5. The results revealed that amoxicillin and rifampicin were the most effective for *S. equi* subsp. *zooepidemicus* followed by streptomycin. The results further revealed that resistance was highest for penicillin G followed by methicillin.

**Table-5:** Results of antibiogram for *S. equi* subsp. *equi* and *S. equi* subsp. *zooepidemicus*.

Antibiotics	*S. equi* subsp. *equi* (N=2)			*S. equi* subsp. *zooepidemicus* (N=12)		
	
	Sensitive isolates (%)	Resistant isolates (%)	Intermediate isolates (%)	Sensitive isolates (%)	Resistant isolates (%)	Intermediate isolates (%)
Amoxicillin	100	0	0	83.33	16.66	0
Penicillin G	0	50	50	16.66	75	8.3
Streptomycin	50	0	50	66.66	16.66	16.66
Rifampicin	100	0	0	75	25	0
Methicillin	0	0	100	0	58.33	41.66

S. equi=Streptococcus equi

## Discussion

Although a variety of bacterial agents have been associated with the respiratory problems in equines, the most important ones which are associated with upper respiratory tract diseases include streptococci (*S. equi* subsp. *equi* and *S. equi* subsp. *zooepidemicus*) [18]. During our study, isolation of streptococci remained standard for the diagnosis of these bacteria but due to the fastidious nature and time involved in the diagnosis, by routine culturing, PCR was attempted for detection of these bacteria directly from the pure isolates. Moreover, PCR assay is more rapid and sensitive [14] than microbiologic culture and is highly specific [19].

In this study, a total of 218 isolates were recovered from 96 samples. When the information generated was compared to the health status of animals, it was found that isolation rate was 121 isolates from 50 samples of diseased animals compared to 97 isolates from 46 samples of apparently healthy animals. Ijaz *et al*. also reported higher prevalence of *S. equi* subsp. *equi* during early spring season only [20]. However, Malik and Kalra did not find any relation of the availability of cases of strangles or other respiratory tract diseases with the season [21]. This may be due to the fact that in their study they selected mostly tropical areas where the effect of temperature variation was not significant. Recently, an outbreak of strangles in 200 horses due *S. equi* subsp. *equi* was reported in the UK that led to significant economic and welfare costs [22].

An outbreak of strangles was reported in the horses working at brick kilns in Jammu, India [23]. They found 38 out of 43 animals to be infected and recorded morbidity of 88.37%. The diagnosis was made by them on the basis of clinical symptoms and microbiological culture of the nasopharyngeal swabs.

Application of the PCR technique was previously described for the detection of *S. equi* subsp. *equi* in nasal and abscess swabs from the horses in a local stud farm and a quarantine station in Malaysia [24]. They used conventional culture method and *SeM* based PCR for the detection of *S. equi* subsp. *equi*. They were unable to detect the *S. equi* subsp. *equi* from any of the samples. The higher prevalence and diversity of *SeM* gene was also reported in strangles outbreak in Brazil [25].

Out of 18 samples were subjected to PCR for detection of *S. equi* subsp. *zooepidemicus* were taken directly from pure isolated colonies, out of which 12 were found to be positive for *sodA* gene. The high recovery rate of this bacterium from the upper respiratory tract of equines has also been reported by Malik and Kalra who obtained 16 isolates of *S. equi* subsp. *zooepidemicus* from a total of 35 isolates of streptococci recovered in their study [21]. Similar findings had been reported by Jannatabadi *et al*. who got 25 isolates of *S. equi* subsp. *zooepidemicus* from 30 cases of respiratory diseases of equines [26]. In contrary to my findings in a recent study where *S. zooepidemicus* was isolated from tracheal washes in only 21% of healthy horses [27].

Thus, it seems from frequent recovery of *S. equi* subsp. *zooepidemicus* from cases of respiratory diseases that it is involved in causing mild respiratory diseases [12] besides *S. equi* subsp. *equi*. Although the role of *S. equi* subsp. *zooepidemicus* as primary bacterial pathogen remains debatable, the most recognize it as the most common bacterial pathogen isolated from equine cases [18]. Mir *et al*. conducted bacteriological and molecular detection of *S. equi* sub sp. *equi* and *S. equi* subsp. *zooepidemicus* in equines of Northern India [28].

A total of 141 samples were collected in duplicate from nasopharyngeal tract of diseased (53) and apparently healthy equines (88) for isolation and direct PCR reported PCR more sensitive than that of routine laboratory culture technique [12]. The reason for less recovery through culture technique could be less number of bacteria present in the mucosal epithelium due to the efficient mucociliary apparatus of equines and rapid desiccation of the bacteria outside the host. Another possible reason responsible for obtaining less number of isolates could be failure of transfer of organism from swab sample to the culture plates. Since the diagnosis of *Streptococcus zooepidemicus*, on the basis of the isolation of *S. equi* subsp. *zooepidemicus* is difficult and time consuming process, not only because of the slow-growing and fastidious nature of this facultative anaerobe, but also because of the overgrowth of a large number of different bacteria. Therefore, we suggest that *sodA* based PCR assay should be used for rapid identification of *S. equi* subsp. *zooepidemicus*. Recently, *sod*A gene characterized by real-time PCR in Uppasala [29].

Growth of *S. equi* grown on sheep blood agar and Columbia nalidixic agar plate was very slow and very difficult to visualize initially, due to the small size of the colonies. However, on confirmation by Gram-staining and PCR analysis, *S. equi* subsp. *equi*, and *S. equi* subsp. *zooepidemicus* were found to be present in the cultures. Thus, the presence of *S. equi* subsp. *equi* and *S. equi* subsp. *zooepidemicus* in the cultures were in agreement with other findings of who reported that Columbia nalidixic agar media improves the growth and facilitates the isolation of the organism [30]. The probable reason can be attributed to the sporadic nature of the disease [31]. The importance of *S. equi* subsp. *zooepidemicus* as a cause of upper respiratory disease with the potential to be transmitted between horses and cause outbreaks is currently being investigated [32].

To find the of drug resistance of bacteria, all the *S. equi* isolates were sensitive to amoxicillin and streptomycin. Most of the isolates were resistant to penicillin G and methicillin. Most of the *S. equi* subsp. *zooepidemicus* isolates were sensitive to amoxicillin and Streptomycin and resistant to penicillin G streptococcal isolations. Earlier reports of the antimicrobial sensitivity on streptococcal isolates also indicated a wide variation in the sensitivity to various antibiotics used [33,34]. Resistance of the some of the isolates to a number of antibiotics seems to be outcome of indiscriminate use of those antibiotics in the field. The antibiogram study indicates that amoxicillin and streptomycin are the effective drugs used against bacterial pathogens. However, the emergence of drug resistance bacteria can be alarming which needs close and repeated vigilance. The indiscriminate use of antibiotics should be avoided. The different combination of antibiotics should be used from time to time.

## Conclusion

From this study, it was concluded that the majority of streptococcal infections were due to *S. equi* subsp. *Zooepidemicus*, and thus was recognized as a potential pathogen of diseases of equines besides *S. equi* subsp. *equi*. These pathogens were detected directly by *SeM*, *sodA*, and *16SrRNA* (species specific) gene amplification. Furthermore, it was found that indiscriminate use of the antibiotics is leading toward the development of resistant strains of *Streptococcus*.

## Authors’ Contributions

RJ and AKT designed the study. Laboratory work was done by RJ and AKT. RJ, AKT, RG and RKS all the authors participated in data analysis, while RJK and RG have drafted and revised the manuscript. All authors read and approved the final manuscript.
